# Consensus on the Clinical Use of Pregabalin in Peripheral Neuropathic Pain

**DOI:** 10.1002/cns.70866

**Published:** 2026-06-17

**Authors:** Hui Lu, Yuefeng Rao, Jian Wang, Wan Shuanglin, Du Dongping, Ma Ke, Zhiying Feng, Suming Tian, Weidong Qiu, Xu Jing, Zhongwei zhao, Zhenhua Zeng, Benyan Luo, Changqing Wang, Hui Zhao, Kaiming Liu, Li Lin, Xianfeng Zhang, Gong Liyan, Haiqi Lu, Yan Liu, Qingwei Zhao, Yihe Hu

**Affiliations:** ^1^ Department of Orthopedics, The First Affiliated Hospital Zhejiang University School of Medicine Hangzhou Zhejiang China; ^2^ Department of Clinical Pharmacy, The First Affiliated Hospital Zhejiang University School of Medicine, Zhejiang Provincial Key Laboratory for Drug Evaluation and Clinical Research Hangzhou Zhejiang China; ^3^ Department of Wound Healing The First Affiliated Hospital of Wenzhou Medical University Wenzhou China; ^4^ Department of Orthopedics Zhejiang University School of Medicine Sir Run Run Shaw Hospital Hangzhou China; ^5^ Department of Pain Management Shanghai Sixth People's Hospital Shanghai China; ^6^ Department of Pain Medicine Shanghai Ninth People's Hospital, Shanghai Jiao Tong University School of Medicine Shanghai P. R. China; ^7^ Department of Pain Medicine, The First Affiliated Hospital Zhejiang University School of Medicine Hangzhou Zhejiang China; ^8^ Department of Anesthesia and Pain Medicine Zhejiang University School of Medicine Sir Run Run Shaw Hospital Hangzhou China; ^9^ Department of Anesthesia and Pain Medicine, The Second Affiliated Hospital Zhejiang University School of Medicine Hangzhou China; ^10^ Department of Pain Management Shanghai Pudong New Area Gongli Hospital Shanghai P. R. China; ^11^ Department of Pain Medicine, and Key Laboratory of Imaging Diagnosis and Minimally Invasive Intervention Research Fifth Affiliated Hospital of Wenzhou Medical University Lishui Wenzhou China; ^12^ Pain Department The First People's Hospital of Jiashan Jiaxing Zhejiang China; ^13^ Department of Neurology The First Affiliated Hospital, Zhejiang University School of Medicine Zhejiang Hangzhou China; ^14^ Department of Neurology First Affiliated Hospital of Anhui Medical University Hefei Anhui China; ^15^ Department of Neurology Affiliated Drum Tower Hospital of Medical School, Nanjing University Nanjing Jiangsu P. R. China; ^16^ Department of Neurology, the Second Affiliated Hospital Zhejiang University School of Medicine Hangzhou China; ^17^ Department of Endocrinology Zhejiang University School of Medicine Sir Run Run Shaw Hospital Hangzhou China; ^18^ Department of Endocrinology, Affiliated Hangzhou First People's Hospital Zhejiang University School of Medicine Hangzhou China; ^19^ Department of Pain & Rehabilitation Medicine, Zhejiang Cancer Hospital Hangzhou Institute of Medicine (HIM), Chinese Academy of Sciences Hangzhou Zhejiang China; ^20^ Department of Internal Medicine‐Oncology Zhejiang University School of Medicine Sir Run Run Shaw Hospital Hangzhou China; ^21^ Department of Dermatology, Jiangsu Province Hospital of TCM Affiliated Hospital of Nanjing University of TCM Nanjing China

**Keywords:** adverse reactions, cancer pain, diabetic peripheral neuralgia, peripheral neuropathic pain, postherpetic neuralgia, postoperative chronic neuralgia, post‐traumatic neuralgia, pregabalin

## Abstract

**Background:**

Peripheral neuropathic pain (pNP) is a prevalent and complex clinical condition that presents significant therapeutic challenges, with pharmacological options demonstrating variable efficacy and safety.

**Objective:**

To establish expert consensus recommendations on the clinical use of pregabalin in the management of diverse pNP conditions.

**Methods:**

A multidisciplinary panel of Chinese experts from orthopedics, pain management, neurology, oncology, dermatology, and pharmacy conducted a comprehensive literature review and synthesized clinical evidence to formulate consensus‐based recommendations.

**Results:**

Pregabalin, a second‐generation calcium channel regulator, demonstrated broad efficacy across multiple pNP conditions, including postherpetic neuralgia (PHN), diabetic peripheral neuropathy (DPN), neuropathic cancer pain (NCP), postoperative and post‐traumatic neuralgia, and trigeminal neuralgia (TN). In PHN, pregabalin significantly reduced pain intensity and improved sleep quality. In DPN, it provided consistent analgesia with improved pain scores and good tolerability. In NCP, pregabalin effectively alleviated pain, showed no significant drug–drug interactions, and was safely co‐administered with chemotherapy. It was also effective in postoperative and post‐traumatic neuralgia, although caution is warranted in cases of traumatic neuroma. In TN, pregabalin exhibited favorable low‐dose efficacy, particularly in elderly patients, with minimal adverse effects. However, cautious use is advised in patients with nerve compression syndromes, such as carpal tunnel syndrome, and in individuals with a history of substance abuse.

**Conclusions:**

This consensus highlights pregabalin as an effective and generally well‐tolerated therapeutic option for a broad range of pNP conditions. These recommendations provide clinically relevant guidance for optimizing pregabalin use, particularly within the Chinese healthcare context.

## Graphical abstract

Pregabalin, a second‐generation α2δ calcium‐channel ligand, exerts potent analgesic effects in peripheral neuropathic pain by reducing calcium influx, suppressing excitatory neurotransmitter release, and decreasing neuronal hyperexcitability. Based on a PRISMA‐guided review of 139 studies and multidisciplinary expert consensus, pregabalin demonstrates robust efficacy across major pNP conditions, including postherpetic neuralgia, diabetic peripheral neuropathy, cancer‐related neuropathic pain, postoperative/post‐traumatic neuralgia, and trigeminal neuralgia. Clinical benefits include rapid pain relief, improved sleep and anxiety, high bioavailability, and minimal drug–drug interactions. Cautious use is recommended in nerve compression syndromes and individuals at risk of drug misuse.

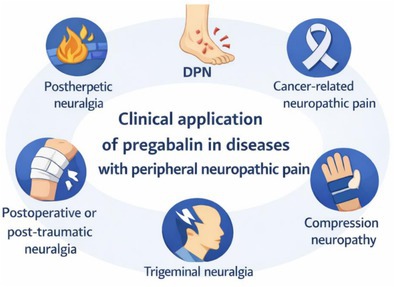

## 1 Introduction

**FIGURE 1:**
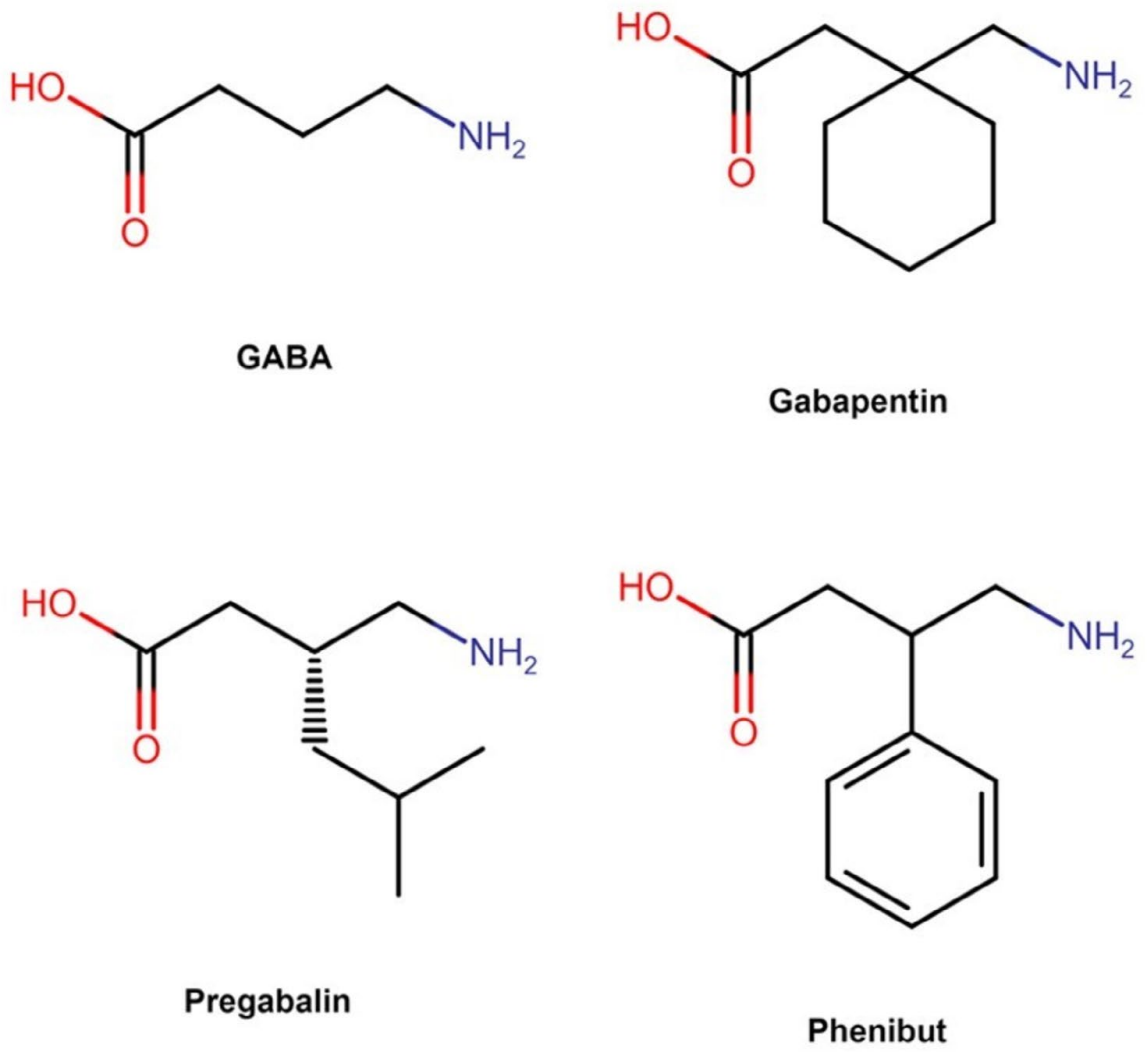
Structural comparison between the inhibitory neurotransmitter gamma‐aminobutyric acid and its analogs gabapentin, pregabalin, and phenibut.

Pregabalin [(S)‐3‐aminomethyl‐5‐methylhexanoic acid, C₈H₁₇NO₂] is a structural analogue of gamma‐aminobutyric acid (GABA) that exhibits anticonvulsant, analgesic, and anxiolytic properties (Figure 1) [1]. As a gabapentinoid, pregabalin functions primarily by binding with high affinity to the α2δ‐1 and α2δ‐2 auxiliary subunits of voltage‐dependent calcium channels (VDCCs), thereby inhibiting calcium influx and modulating neurotransmitter release [2, 3]. Notably, in contrast to GABA and classical GABA‐receptor agonists, pregabalin does not exhibit affinity for GABA receptors and is not metabolically converted to GABA, underscoring its distinct mechanism of action. Instead, it induces a dose‐dependent upregulation of L‐type amino acid transporters, such as those for L‐leucine, L‐valine, and L‐phenylalanine, which are implicated in the endogenous synthesis of GABA in the central nervous system (CNS) [3, 4, 5, 6]. Recently, pregabalin was found to interact only with the molecular site α2δ‐1, which was also believed as the only relevant functional site of pregabalin. Clinical indications of pregabalin included anxiety disorders, pain disorders, epilepsy, and others [7]. Among various indications, peripheral neuropathic pain (pNP) was highlighted in this analysis to reach a consensus. Neuropathic pain is manifested by damage to the nervous system, differing from the pain that is carried along healthy nerves from damaged tissue (pain from a fall or a cut, or an arthritic knee) [8] The widely accepted current definition of pNP was the pain caused by a lesion or disease in the somatosensory system [9]. The definition included but was not limited to the pain invoked by postherpetic neuropathy, diabetic neuropathy, nerve damage by malignant tumors and treatment, and traumatic neuromas. The prevalence of neuropathic pain could be as high as 7%–8% in the general population, hindering social and economic development [10, 11]. The review gathers insights from a multidisciplinary panel of Chinese experts in various fields including orthopedics, pain management, neurology, oncology, dermatology, and pharmacy. Their collective expertise, coupled with a comprehensive review of the literature, forms the basis for developing a series of recommendations. These recommendations are aimed at highlighting pregabalin's effectiveness in treating a spectrum of pNP conditions. The conditions discussed include postherpetic neuralgia (PHN), diabetic peripheral neuralgia (DPN), cancer pain, and others, with a particular focus on the implications for the Chinese healthcare context. The consensus also addresses the pharmacological aspects of pregabalin, including its mechanism of action as a second‐generation calcium channel regulator. It delves into the clinical indications of pregabalin, not just limited to pNP but also encompassing areas such as anxiety disorders and epilepsy. The review critically appraises various studies and trials, providing a well‐rounded perspective on pregabalin's utility in clinical practice (Table 1).

**TABLE 1:** Summary of clinical application of pregabalin in diseases with peripheral neuropathic pain.

Study	Population	Design	Dosage	Findings
Mercan et al. [12]	Postherpetic neuralgia (PHN) patients, *n* = 40	Single‐arm observational	Pregabalin, 3 months	Significant increase in CD4^+^ and Th17 immune cells; pregabalin may modulate immune pathways involved in neuropathic pain.
Baron et al. [13]	PHN (*n* = 96) and DPN (*n* = 204) patients	Open‐label, non‐inferiority RCT	Flexible, titrated pregabalin	Pregabalin effective in DPN (69.1% response); less effective in PHN (46.5%) than lidocaine plaster; both improved allodynia and QoL.
Rosenstock et al. [14]	Diabetic peripheral neuropathy (DPN) patients, *n* = 146	8‐week randomized placebo‐controlled trial	Pregabalin 300 mg/day	Significant reductions in pain (*p* < 0.0001), sleep interference, mood disturbance, and improved QoL compared to placebo.
Richter et al. [15]	DPN patients, *n* = 246	6‐week randomized controlled trial	Pregabalin 150 or 600 mg/day	600 mg/day group showed significant pain reduction (mean pain score 4.3 vs. 5.6 placebo); 39% had ≥ 50% pain reduction; improved sleep.
Freynhagen et al. [16]	PHN or DPN patients, *n* = 338	12‐week multicenter RCT	Flexible (150–600 mg/day) or fixed 600 mg/day	Both regimens significantly reduced mean pain and sleep interference compared to placebo; well tolerated.
Satoh et al. [17]	Japanese DPN patients, *n* = 317	14‐week randomized controlled trial	Pregabalin 300 or 600 mg/day	Significant reduction in pain from Week 1, sustained throughout; 35.6% (600 mg) achieved ≥ 50% pain reduction; improved sleep and QoL.
Mishra et al. [18]	Neuropathic cancer pain patients, *n* = 120	Double‐blind, placebo‐controlled RCT	Pregabalin vs. amitriptyline vs. gabapentin	Pregabalin was the most effective in pain reduction, symptom control, and quality‐of‐life improvement; showed morphine‐sparing effect.
Singh et al. [19]	Post‐traumatic nerve injury (facial), *n* = 2 (case series)	Case study	Pregabalin (dose not specified)	Effective in treating facial neuropathic pain unresponsive to analgesics; showed good tolerability.
Hamasaki et al. [20]	Refractory trigeminal neuralgia, *n* = 33	Retrospective observational study	Low‐dose pregabalin pre‐surgery	48.5% had pain improvement; most effective in elderly patients (age > 62.7 predicted better response); useful pre‐surgery salvage option.
Obermann et al. [21]	53 TN patients (14 with facial pain)	Open‐label, 1 year follow‐up	150–600 mg/day (mean 270 mg)	74% improved; better response without facial pain; 42% had side effects.
Pérez et al. [22]	65 PGB‐naive, refractory TN patients	12‐week observational (primary care)	196 mg/day (mono), 234 mg/day (add‐on)	59% had ≥ 50% pain reduction; better outcomes with monotherapy.
Rustagi et al. [23]	22 CBZ‐refractory TN patients	Randomized crossover (vs. LTG + CBZ)	Mean 241 mg/day	PGB = LTG in efficacy; fewer side effects and better compliance.

### 1.1 Literature Evaluation Methodology

**FIGURE 2:**
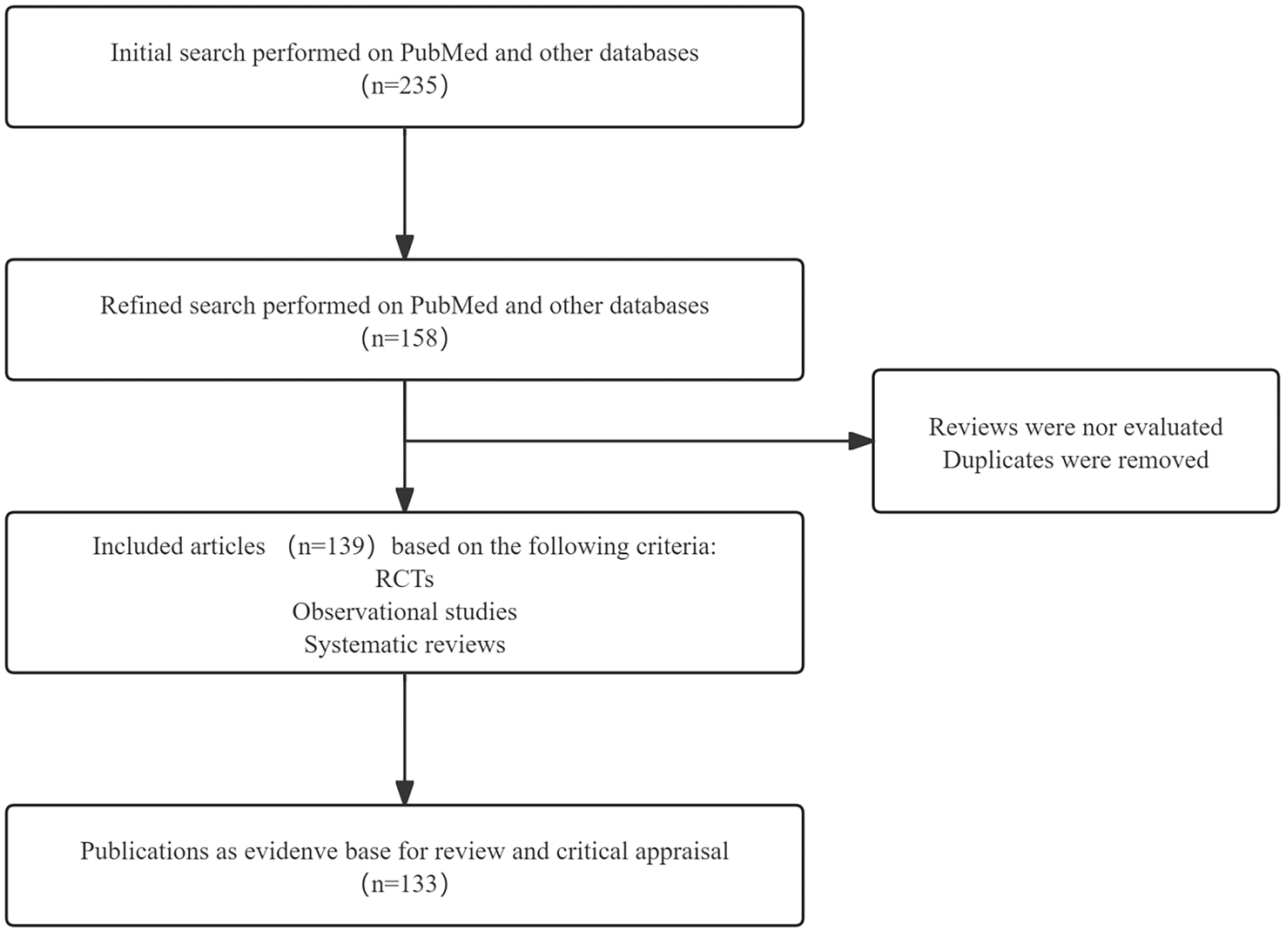
Preferred reporting items for systematic reviews diagram of relevant literature identified. RCTs, randomized controlled trial.

A systematic literature review was conducted in accordance with the Preferred Reporting Items for Systematic Reviews and Meta‐Analyses (PRISMA) guidelines to support the development of this expert consensus on the clinical application of pregabalin in peripheral neuropathic pain (pNP). Literature was sourced from five major biomedical databases: PubMed, Embase, Web of Science, Cochrane Library, and the China National Knowledge Infrastructure (CNKI). The search strategy employed a combination of Medical Subject Headings (MeSH) and free‐text keywords using Boolean operators. Core terms included “pregabalin” OR “Lyrica” AND “neuropathic pain,” “peripheral neuropathy,” “postherpetic neuralgia,” “diabetic neuropathy,” “cancer pain,” “chemotherapy‐induced peripheral neuropathy,” “trigeminal neuralgia,” “nerve compression,” and “traumatic neuroma.” The review also incorporated gray literature, including clinical trial registries (e.g., ClinicalTrials.gov), relevant conference abstracts, and the reference lists of pertinent systematic reviews to ensure comprehensive coverage and minimize publication bias. The initial search yielded 235 records. After removing duplicates, two independent reviewers screened titles and abstracts based on predefined eligibility criteria. Full texts of 158 potentially relevant studies were assessed, and 139 articles were ultimately included in the qualitative synthesis (Figure 2). Study inclusion was guided by the PICO (Population, Intervention, Comparator, Outcome) framework. Eligible studies involved patients diagnosed with peripheral neuropathic pain, including conditions such as postherpetic neuralgia, diabetic peripheral neuropathy, cancer‐related nerve injury, postoperative or traumatic neuralgia, trigeminal neuralgia, and nerve compression syndromes. Interventions of interest focused on pregabalin, either as monotherapy or in combination with other treatments. Comparators included placebo or alternative pharmacological agents such as gabapentin, antidepressants, opioids, or topical agents. Primary outcomes included pain reduction, improvement in sleep quality, functional status, and adverse event profiles. Eligible study types included randomized controlled trials (RCTs), cohort studies (prospective or retrospective), systematic reviews, and meta‐analyses. Case reports, narrative reviews, and editorials were excluded. Studies in languages other than English or Chinese were excluded unless a high‐quality English translation was available. Conference abstracts were included only if sufficient methodological detail and outcome data were provided. To ensure methodological rigor, the quality of included studies was critically appraised. RCTs were assessed using the Cochrane Risk of Bias Tool, while observational studies were evaluated using the Newcastle‐Ottawa Scale. Two reviewers independently conducted risk of bias assessments, with discrepancies resolved through consensus or consultation with a third reviewer. Levels of evidence and grades of recommendation were assigned according to the Oxford Centre for Evidence‐Based Medicine (OCEBM) 2011 framework. Evidence quality was categorized as Level I (high), II (moderate), III (low), or IV (very low), and strength of recommendation was graded as A (strong), B (moderate), or C (not recommended) (Table 2). These designations were applied consistently throughout the manuscript to support the clinical guidance statements. The complete study selection process is detailed in the PRISMA flow diagram.

**TABLE 2:** Representations of quality of evidence and strength of recommendation.

Quality of evidence	
High quality	I
Moderate quality	II
Low quality	III
Very low quality	IV
Strength of recommendation	
Strong recommendation for intervention	A
Weak recommendation for intervention	B
Not recommended for intervention	C

### 1.2 Mechanism of Action

Pregabalin was the first agent approved by the U.S. Food and Drug Administration (FDA) for the treatment of diabetic peripheral neuropathy and postherpetic neuralgia, supported by extensive preclinical and clinical evidence [24]. The target receptor for pregabalin is α2δ‐2 subunit on certain VDCCs, which shows an increase in expression on the dorsal root ganglion (DRGs) and dorsal horn in patients suffering from neuropathic pain [25]. Pregabalin is a calcium channel blocker (CCB). Studies found that VDCCs expressing α2δ subunits are rate‐limiting channels in synaptic modulation compared with VDCCs expressing α1 subunits. Moreover, in animal studies, mice overexpressing α2δ‐1 displayed neuropathic phenotypes of hyperalgesia and tactile allodynia even in the absence of nerve injury, indicating that α2δ‐1 overexpression led to abnormal excitability of DRGs and neuropathies [26]. Pregabalin was also involved in trafficking excitatory amino acid transporters (EAATs) from the cytosol to the plasma membrane in a concentration‐dependent manner, which was hindered by PKC and P13K inhibitors, indicating the possible signaling target by these molecules [27, 28]. Additionally, a study led by Huang et al. in 2006 showed that pregabalin activated K_ATP_ channels, describing another potential mechanism for its analgesic effects [29]. Another study observed that whether targeted inside or outside the DRGs, pregabalin stimulated an enhanced K^+^ current, suggesting the presence of intracellular and extracellular target sites [30]. Consequently, enhanced K^+^ current decreased neuronal excitability and ameliorated the symptoms of neuropathic pain [31]. However, it should be noted that as a controlled substance, although the potential risk of pregabalin abuse is lower than that of other opioid analgesics, it is not to be overlooked as put forward by Schjerning O. et al. in a systematic review, where they discovered an important clinical abuse potential of pregabalin [32].

In general, pregabalin exerts analgesic function potentially via targeting α2δ‐1 and α2δ‐2 subunits on VDCCs to regulate Ca^+^ current, targeting EAAT‐trafficking to limit signaling via excitatory neurotransmitters including glutamate and enhancing K^+^ current to decrease neuronal excitability. It can be concluded that pregabalin functions via various mechanisms and produces a potent analgesic effect in patients suffering from neuropathic pain.

### 1.3 Pharmacokinetics

After oral administration, the bioavailability of pregabalin was equal to or greater than 90% but affected by food intake [33]. Normally, food can delay or decrease its absorption, when the *T*_max_ is prolonged up to 3.2 h compared with 0.6 h in fasting conditions, and *C*_max_ shows a 25%–31% reduction from the fasted state where the bioavailability remains unchanged [33]. Upon absorption, pregabalin is actively transported across the blood–brain barrier (BBB) via L‐type amino acid transporter 1 (LAT1), and the volume of distribution in humans is approximately 0.56 L/kg [34]. Pregabalin does not metabolize in the liver and does not bind to plasma proteins, resulting in nearly 98% renal clearance and excretion of unchanged pregabalin [35].

### 1.4 Drug Interactions

Pregabalin is characterized by complete absorption from the gastrointestinal tract, minimal hepatic metabolism, and predominant renal excretion in its unchanged form. Therefore, it demonstrates a low potential for pharmacokinetic interactions with other medications. However, certain clinically relevant pharmacodynamic interactions have been documented and warrant caution. Notably, the co‐administration of pregabalin with central nervous system (CNS) depressants, particularly opioids, may lead to additive effects such as dizziness, sedation, confusion, and cognitive impairment. In severe cases, this combination has been associated with life‐threatening respiratory depression, especially in elderly or debilitated patients. For example, co‐administration with oxycodone has been linked to psychotic reactions in rare instances, requiring dosage adjustment or discontinuation of therapy [36]. When concurrent use with opioids is unavoidable, it is recommended to reduce the opioid dose by approximately 50% and to monitor patients closely for signs of respiratory compromise. Similarly, concurrent use of pregabalin with benzodiazepines may exacerbate CNS depression, resulting in excessive sedation, profound drowsiness, and respiratory depression due to cumulative inhibitory effects on the brainstem respiratory centers. Other agents with sedative properties, including first‐generation antihistamines, muscle relaxants, sedatives, and antipsychotics such as clozapine, can potentiate pregabalin's depressant effects. In particular, elevated serum clozapine concentrations and increased risk of postural instability have been reported following co‐administration with pregabalin, likely due to synergistic pharmacodynamic interactions [37, 38]. Therefore, a cautious and individualized approach to polypharmacy is recommended, particularly in populations at higher risk for CNS and respiratory depression, such as the elderly or those with underlying cardiopulmonary conditions.

### 1.5 Summary of Pharmaceutical Properties

As a second‐generation calcium channel regulator, pregabalin has obvious advantages over the first‐generation calcium channel regulator plus gabapentin. It exerts stronger affinity to α2‐δ subunits of VDCCs in the CNS, faster titration and onset of action, linear pharmacokinetic characteristics, and with ≥ 90% bioavailability. Pregabalin achieves its analgesic effect by regulating the influx of calcium ions, reducing the excessive release of excitatory neurotransmitters, and inhibiting central sensitization. The drug interaction is low since it is completely excreted by the kidney, except for the additive side effects with some other drugs [39, 40].

## 2 Clinical Application of Pregabalin in Diseases With Peripheral Neuropathic Pain

### 2.1 Postherpetic Neuralgia

Postherpetic neuralgia (PHN) is a neuropathic pain syndrome that occurs as a complication of herpes zoster, diabetes mellitus, and malignant cancers [41, 42, 43]. This condition is characterized by persistent neuropathic pain that endures for months or even years following the resolution of the acute herpes zoster rash. The condition results from reactivation of the varicella‐zoster virus, which causes direct injury to sensory nerves in the affected dermatomes [44]. Clinically, PHN presents with chronic pain, allodynia (pain due to stimuli that do not normally provoke pain), and sensory deficits localized to the involved dermatome. In some patients, the pain may persist for years, significantly impairing daily functioning and quality of life. Notably, over 80% of individuals with PHN report psychiatric comorbidities such as depression and anxiety, underlining the need for holistic management approaches [45]. Preventing PHN primarily involves the timely treatment of herpes zoster and vaccination. Once PHN develops, various treatment options are available, including antiepileptics, antidepressants, local lidocaine, and other pain management strategies [46, 47]. The immune system plays an important role in the development and management of neuropathic pain and some immunological marker levels are different before and after the treatment of postherpetic neuralgia. The immunological effect of pregabalin in patients with PHN was investigated and studies found that Th17+ and CD4T+ cells increased in number while the number of Treg cells remained unchanged. Additionally, the long‐term pregabalin treatment significantly decreased the c‐reactive protein (CRP) and erythrocyte sedimentation rate (ESR), shortening the acute inflammatory period due to the positive correlation of CRP with acute inflammation, therefore decreasing PHN [48]. A previous study found that a low CD4T cell count was a high‐risk factor that caused PHN, but pregabalin treatment prevented this risk by increasing the CD4T cell counts and decreasing neuropathic pain symptoms [12]. After pregabalin treatment, T cells were almost completely recovered, thus alleviating PHN [12]. Therefore, pregabalin has been recommended to be the first‐line pharmacological agent in treating PHN. Economic considerations have also been evaluated. Fei et al. compared the cost‐effectiveness of various first‐line treatments for PHN in China, reporting that 5% lidocaine medicated plasters (LMPs) offered superior safety, efficacy, and quality‐of‐life improvements compared to pregabalin at doses of 300 mg and 488 mg/day. Additionally, incorporating LMPs into the national reimbursement system resulted in a significant reduction in healthcare expenditures [13, 49]. However, in a multinational clinical trial, Parsons et al. demonstrated that pregabalin was equally effective and well‐tolerated in both Chinese and international populations. In addition, pregabalin reduced pain and improved the sleep pattern more than LMP, the mechanism is not clearly known but could be due to its high binding capability to the neuronal voltage gated channels, and the improvement of anxiety symptoms. Further, pregabalin had a faster effect on pain relief compared with the placebo group, and no significant adverse effects that distinguish Chinese from international patients were found [50]. The effective dosages of pregabalin approved by the FDA were 300–600 mg/day as a flexible dosing regimen [51]. Even with different dosages, the onset of pain relief and improvement in PRSI scores were evident within 2 days compared with 18 days by placebo [52, 53].

#### 2.1.1 Recommendation 1

Pregabalin can effectively reduce pain symptoms and improve sleep patterns in patients with PHN, with a good safety level (I A).

### 2.2 Painful Peripheral Neuropathy in Diabetes

Diabetic peripheral neuropathy (DPN) is a common complication associated with type 1 and type 2 diabetes, and normally the nerves in the feet and hands are damaged. The development of DPN involves multiple metabolic pathways, including the activation of the polyol pathway, the hexosamine pathway, protein kinase C pathway, and the production of AGEs. Growth factors related to the synthesis and apoptosis of Schwann cells also play a crucial role [54]. The clinical manifestations of DPN vary depending on the extent and type of nerve involvement. Common symptoms include paresthesia, burning or stabbing pain, numbness, and motor weakness. Epidemiological data indicate that approximately 40% of patients with diabetes develop DPN, and its prevalence and severity increase with poor glycemic control and disease duration [55, 56]. Notably, nearly 70% of individuals with DPN experience chronic and severe neuropathic pain, which significantly impairs quality of life and is often accompanied by insomnia, depression, suicidal ideation, personality changes, and a substantial economic burden related to ongoing treatment and management [57]. Management of DPN focuses on symptom relief and prevention of further nerve deterioration. Optimal glycemic control remains a cornerstone of therapy, as it slows the progression of neuropathy and improves clinical outcomes. Pharmacologic pain management includes tricyclic antidepressants, serotonin‐norepinephrine reuptake inhibitors (SNRIs), and anticonvulsants such as pregabalin and gabapentin, which have demonstrated efficacy in reducing neuropathic pain [58, 59]. Furthermore, multiple RCTs and systematic reviews have demonstrated the efficacy of pregabalin in the management of DPN. It is recommended as a first‐line therapeutic agent in several international clinical guidelines due to its proven benefits in pain reduction, improved tolerability, and enhanced patient‐reported outcomes [56, 60, 61, 62]. In addition to its analgesic properties, pregabalin has been shown to alleviate comorbid conditions such as anxiety and sleep disturbances commonly associated with DPN [56]. A study reported that pregabalin at a dose of 300 mg/day for 8 weeks noticeably improved pain scores, sleep interference, and anxiety [14]. Another trial that included 338 participants found that 300 mg/day and 600 mg/day pregabalin dosages for 5 weeks also improved the pain scores and reduced the sleep disturbance, but the 600 mg/day dosage showed a better percentage of pain relief more than 48% compared with 45% by 300 mg/day dosage [63]. Moreover, a 6‐week randomized controlled trial that included 246 patients with DPN showed that 600 mg/day pregabalin reduced the pain scores significantly, by more than 50% compared with lower doses and placebo [15, 16]. Rainer et al. reported the superior efficacy of pregabalin at a dosage of 600 mg/day in a multi‐center trial (*n* = 338) [16]. Several clinical trials investigated the efficacy of pregabalin in patients with DPN and PHN specific to pain relief and tolerability. Meta‐analyses on pregabalin treatment with different cohorts of patients with DPN were also accomplished [64, 65, 66]. The meta‐analyses reported 150 mg/day or higher pregabalin as the effective dose; also, other confirmatory studies showed that the lower than 150 mg/day pregabalin did not have any significant effect [17].

As with all other medications, pregabalin exerted a few mild‐to‐moderate adverse effects, including somnolence, dizziness, peripheral edema, and rarely weight gain. The current explanation of adverse effects referred to the pregabalin mechanism, which influenced the release of neurotransmitters and calcium; therefore, low metabolism rate, increase water retention, and high sedation effect [67]. Weight gain related to pregabalin treatment should be taken as a serious issue in type 2 diabetes due to the risk of deteriorating metabolism. Therefore, randomized controlled trials analyzed the effect of pregabalin with different doses (150–600 mg/day) on lipid or glycaemic profiles in patients with DPN and found no significant effect [68].

#### 2.2.1 Recommendation 2

There are significant clinical benefits associated with pregabalin in patients with DPN, including stable pain relief, better pain scores and tolerability levels, and anxiety and sleep disturbances (I A).

### 2.3 Pain and Peripheral Neuropathy Induced by Cancers and Their Treatments (Chemotherapy, Radiotherapy, and Surgery)

Neuropathic cancer pain (NCP) is a complex and heterogeneous condition encompassing multiple etiologies, each with distinct pathophysiologic mechanisms, clinical characteristics, and therapeutic implications. The IASP and EAPC recommend classifying NCP into three main categories; tumor‐related, treatment‐related, and mixed neuropathic pain. Patients with advanced‐stage malignancies frequently experience moderate to severe chronic pain, which may persist for months or even years. NCP is commonly associated with hallmark neuropathic symptoms such as tingling sensations, electric shock‐like pain, numbness accompanied by muscle weakness, and allodynia [69, 70]. A meta‐analysis that investigated the etiologies of NCP found that 64% of NCP were caused by tumors and 20% by cancer treatments [71]. Oosterling et al. performed a cross‐sectional study and reported that approximately 23% of patients with NCP complained of moderate to severe pain, and around 19% experienced other neuropathic symptoms [72]. Therefore, life quality and daily activities were significantly affected in cancer patients with NPC compared to those without NPC [73]. Preventing and treating NCP are difficult, thus reducing the pain and neuropathic symptoms is mainly focused. In the past several years, patients with NCP have used various drugs. Serotonin‐norepinephrine reuptake inhibitors (SNRIs) (duloxetine and venlafaxine) and gabapentinoids (gabapentin and pregabalin) were the first‐line treatments, according to the American society of clinical oncology guidelines [74]. The National Comprehensive Cancer Network (NCCN) guidelines and World Health Organization (WHO) recommend the combination of opioids and gabapentinoids to enhance pain relief. Gabapentin and pregabalin are new anticonvulsants with fewer adverse effects than the old‐generation drugs, such as valproic acid and carbamazepine. Several clinical studies reported the high efficacy of gabapentin against NCP, either alone or with opioids, while pregabalin efficacy was less reported. Additionally, the chronic pain associated with oxaliplatin therapy was not alleviated by pregabalin [75, 76]. Mishra et al. showed that pregabalin was more effective with a low dosage of opioids than gabapentin and amitriptyline through a controlled clinical trial [18]. Generally, gabapentinoids reduce sleep disturbances and anxiety, enhance opioid effectiveness, and assist patients who require opioid reduction. The maximal daily dosages of pregabalin and gabapentin for patients with NCP were different, 600 mg/day and 3600 mg/day, respectively, and because of its linear pharmacokinetics and dose‐dependent absorption characteristics, it reduced the pain more rapidly with higher potency than gabapentin [76]. In addition, patients who were exposed to chemotherapy or cytotoxic agents such as taxanes, anti‐tubulins, and alkaloids experienced peripheral neuropathy as an adverse effect with an incidence of less than 7% if exposed to a single cytotoxic agent or about 38% with combination therapy [77, 78]. Moreover, the most common symptoms associated with chemotherapy‐induced peripheral neuropathy (CIPN) were dysesthesia, paresthesia, hyperalgesia, hypoalgesia, and shooting or burning pain [77, 78]. Furthermore, the severity of CIPN depended on several factors related to the chemotherapy agents, such as the use of multiple agents, duration of exposure, and cumulative dose, as well as coexisting disorders or diseases, for instance, diabetes, alcoholism, and vitamin deficiency (B12 deficiency) [65] Prevention or treatment of CIPN has been investigated and the pathophysiological mechanism of the side effects caused by chemotherapeutic agents caused was well understood. Normally, these agents upregulated the expression of the sodium channel, α2‐δ1 subunit of the calcium channel, and activated N‐methyl‐D‐aspartate [NMDA] receptors, causing a severe influx of extracellular calcium and mitochondrial calcium leakage, and therefore, neural cell death occurs due to increased amount of toxic oxygen radicals and apoptosis activation [17, 79]. Neuromodulators such as pregabalin are effective in treating neuropathic pain by reducing the calcium‐dependent neurotransmitters on the neural membrane, consequently, inhibiting neuronal excitability. Further, the bioavailability of pregabalin is higher than the other neuromodulators, such as gabapentin (90% vs. 66%), and it has no drug interaction, thus, can be safely accompanied by chemotherapeutic agents. The bioavailability of pregabalin does not change by changing the dosage, in contrast with gabapentin bioavailability which deceases by increasing the clinical dose for instance the bioavailability will decreases from 60% to 33% when increasing the gabapentin dose from 900 to 3600 mg/day [39]. Also, pregabalin presents a better efficacy even at lower dosages with a low risk of adverse effects [80, 81].

#### 2.3.1 Recommendation 3

Patients with advanced cancers have moderate to severe pain lasting months or years, and reducing pain and neuropathic symptoms is the primary goal in patients with NCP. As a new generation anticonvulsant, pregabalin is effective in the treatment of neuropathic pain by reducing calcium‐dependent neurotransmitters located on the nerve membrane, is more bioavailable than the other neuromodulators, has no drug interactions, and can be safely used in combination with chemotherapy drugs. (IB).

### 2.4 Postoperative or Post‐Traumatic Neuralgia

Chronic postsurgical pain (CPSP) and chronic post‐traumatic pain (CPTP) are conditions characterized by persistent pain lasting longer than three to 6 months after surgery or traumatic injury, respectively [82, 83, 84]. The incidence and severity of CPSP vary according to the type of surgical intervention and specific procedural techniques. For instance, persistent neuropathic pain is reported in 50%–85% of patients undergoing limb amputation, while lower incidence rates have been observed following thoracotomy, mastectomy, hysterectomy, hip arthroplasty, and colectomy [85]. A study evaluating 3120 postoperative patients found that moderate and severe neuropathic pain occurred in 35.4% and 57.1% of patients, respectively, within 12 months after surgery [86]. Similarly, another longitudinal investigation demonstrated neuropathic pain prevalence rates of 31.9% at 6 months and 40.3% at 12 months postoperatively [87]. CPTP commonly arises following severe tissue trauma, including burns or spinal cord injuries, and frequently manifests as neuropathic pain. The incidence rate of CPTP ranges from 46% to 85%, particularly after multiple traumatic injuries [88]. Post‐traumatic and postsurgical neuropathy can be treated with different types of medications. The first‐line treatments include tricyclic antidepressants, serotonin, gabapentin, and pregabalin. A traumatic neuroma is an abnormal nerve regeneration with different levels of nerve fiber maturation and disorganization as a result of peripheral nerve injuries after a trauma or surgery [89]. The neuroma presented as an entanglement of neural fibers with the surrounding tissue after a nerve injury, and it had a palpable solid elliptical whitish and painful nodule about 2 cm in diameter [90]. Traumatic neuroma remarkably affected the life quality of the patients due to continuous pain, functional weakness, and paresthesia at the site of the neuroma [91]. Medications for traumatic neuroma have different cellular effects. For instance, carbamazepine blocks sodium channels on the neural membrane; therefore, improving the painful paresthesias. Moreover, the new emerging medication in treating traumatic neuroma is pregabalin, with a significant effect in reducing neuropathic pain by blocking the voltage‐dependent calcium channel [19]. Lelio et al. reported a traumatic neuroma on the left infraorbital nerve after an inferomedial orbital decompression operation, and the symptoms did not improve with gabapentin 600 mg twice daily. In contrast, the neuroma has been partially controlled by pregabalin 75 mg three times daily [92]. Several studies mentioned the superiority of gabapentin and pregabalin over other medications, such as lidocaine, antispasmodic and antidepressant drugs, and α receptor blockers in the treatment of traumatic neuroma, due to their long‐term therapeutic effects and minimum adverse effects, through central sensation inhibition [93, 94, 95].

#### 2.4.1 Recommendation 4

Pregabalin can be used for postoperative neuralgia or post‐traumatic neuralgia (IB).

Management of traumatic neuroma should be individualized, taking into account clinical severity and physician expertise (IIB).

### 2.5 Trigeminal Neuralgia

Trigeminal neuralgia (TN), often referred to as tic douloureux, presents with highly distinctive and severe symptoms that significantly impact the quality of life. The hallmark of this condition is its paroxysmal pain, which is both unique and intense [96]. Characteristically, this pain manifests as abrupt, excruciating bursts, often likened to electric shocks or stabbing sensations. These episodes are fleeting, typically lasting from a mere fraction of a second up to 2 min, with most lasting only a few seconds [96]. However, despite their brief duration, they are profoundly distressing for the sufferer. The distribution of pain in TN is restricted to the territories of the trigeminal nerve, predominantly affecting its second (maxillary) or third (mandibular) divisions. A higher incidence on the right side of the face is observed, although the underlying reason for this asymmetry remains unclear. Notably, pain attacks can be triggered by routine activities or mild sensory stimuli, including speaking, facial washing, chewing, or even exposure to gentle airflow. Furthermore, the area of pain occurrence does not always correspond directly with the site of the triggering stimulus [97, 98]. In addition to these paroxysmal episodes, some patients experience a continuous or persistent form of pain, which exists alongside the more characteristic shock‐like episodes. In addition to typical paroxysmal episodes, some patients experience persistent pain alongside characteristic intermittent episodes. This continuous pain, often described as burning, throbbing, or aching, defines a subtype known as trigeminal neuralgia with concomitant continuous pain, highlighting the complexity and multifaceted nature of this disorder [99, 100]. TN can be classified into several categories based on etiology. Classical TN, the most common form, results primarily from vascular compression of the trigeminal nerve root [101]. Secondary TN is associated with identifiable neurological conditions such as multiple sclerosis (MS) or intracranial tumors [102, 103]. Idiopathic TN occurs in the absence of identifiable structural causes, presenting diagnostic and therapeutic challenges. Pharmacological management typically involves sodium channel blockers such as carbamazepine, oxcarbazepine, and lamotrigine, which constitute the first‐line treatment due to their efficacy in controlling paroxysmal pain episodes [104, 105].

Antiepileptic medications, such as lamotrigine, oxcarbazepine, and carbamazepine, are first‐line treatments for TN. These drugs, sodium channel blockers, have shown significant efficacy in managing the sharp, sudden pain characteristic of TN [106]. However, these first‐line treatments are not without drawbacks. A substantial number of patients experience intolerable side effects from these medications, and in some cases, the effectiveness of these drugs can diminish, particularly for those with concomitant continuous pain. In such scenarios, where first‐line treatments either fail to provide adequate relief or cause unacceptable side effects, the consideration of alternative therapeutic options becomes necessary [107]. Pregabalin and other α2δ ligands, which include gabapentin, represent such alternatives. Pregabalin was successful in the treatment of TN. Claudio et al. conducted a clinical study where pregabalin was combined with lamotrigine to treat MS patients, and the combination at small dosages was more effective with minimum side effects [108]. Preoperative treatment of refractory TN with pregabalin was analyzed, and the pain was reduced in 50% of the patients. The effectiveness of pregabalin increased in patients older than 60 years, and this outcome could be due to impaired hepatic and renal function and low body water content. Further, the study recommended the use of pregabalin before the surgery in refractory TN patients, even with a low dose of 150 mg/day [20]. From a review of eleven clinical studies, Semel et al. concluded that 150 mg pregabalin remarkably alleviated the mean pain score in older patients compared with young patients, even with similar dosages [109]. One‐year follow‐up of 53 patients who had refractory TN treated with pregabalin reported that 11 patients among them did not experience pain during the follow‐up period [21]. Another study assessed the effectiveness between monotherapy pregabalin and pregabalin with other medications for 12 weeks in refractory TN patients and found that 39.4% and 21.4% of patients, respectively, presented with free of pain [22]. A further study proved the superiority of pregabalin in TN treatment, in which a 240.68 mg/day dosage completely or partially reduced the pain; in contrast, to a 310.90 mg/day dosage of lamotrigine. Some patients reported relapse of acute symptoms with lamotrigine. Also, the use of pregabalin with carbamazepine provided better pain control than with lamotrigine [23].

Pregabalin shows low interactions with other drugs and few adverse effects, and a low dose can be effective compared with other drugs. All these advantages make pregabalin the preferable treatment for TN patients, especially older patients who suffer from systemic diseases [110].

#### 2.5.1 Recommendation 5

Pregabalin is used to treat patients with TN with few side effects, fewer drug interactions, and significant pain relief at low dosages (150 mg/day) compared to similar drugs, especially in elderly patients (IA).

### 2.6 Pain by Chronic Peripheral Nerve Compression Disease

Chronic peripheral nerve compression, also known as compression neuropathy, is a pathological condition resulting from sustained mechanical pressure on peripheral nerves. This pressure may arise internally, such as from soft tissue masses, or externally, due to trauma or anatomical entrapment. Affected regions typically exhibit motor weakness, sensory disturbances, and neuropathic pain, including numbness, tingling, and burning sensations [94, 111, 112]. Carpal tunnel syndrome (CTS) most frequently occurs as a peripheral nerve entrapment syndrome due to compression of the median nerve at the wrist by different factors such as tendon inflammation, daily activities, and hormonal changes [113]. The cubital syndrome is another example of compression neuropathy in which the ulnar nerve is entrapped within the cubital tunnel at the elbow [111, 112]. Moreover, the radial, peroneal, and lateral femoral cutaneous nerves are entrapped. Additionally, disc herniation compresses the nerve roots of the dorsal ganglia, leading to chronic lower back pain [114]. Management of compression neuropathy is guided by the severity of nerve involvement. Conservative approaches, such as physiotherapy, manual therapy, and nonsteroidal anti‐inflammatory drugs (NSAIDs), are typically first‐line treatments. In more severe or refractory cases, surgical decompression may be required and is often associated with superior clinical outcomes compared to non‐surgical interventions [115]. Although pregabalin shows significant effectiveness in the treatment of pNP, it displays a negative outcome in compression neuropathies such as CTS. Several trials evaluated the efficacy of pregabalin and placebo in compression neuropathies and found no advantages of pregabalin over placebo. Also, preoperative use of pregabalin for carpal tunnel syndrome increases the danger from long‐term gabapentinoid and opioid use [116, 117].

#### 2.6.1 Recommendation 6

Pregabalin may be considered as an adjunctive option in the management of nerve compression syndromes such as CTS; however, it should not be prioritized as first‐line therapy. Topical analgesics, particularly lidocaine patches, should be emphasized within a multimodal pain management strategy to minimize systemic exposure and reduce adverse effects (IIIC).

## 3 Clinical Recommendation of Pregabalin

### 3.1 Routine Usage and Dosage

The dosage and thus efficacy of pregabalin depend on the medical condition and concurrent medications administered. Patients who are sensitive to other medications at low doses may similarly experience adverse effects from even small doses of pregabalin. Therefore, to minimize side effects and achieve maximum therapeutic benefit, it is recommended to initiate treatment with 75 mg orally once daily at bedtime, or 75 mg divided into two daily doses. The dosage can then be gradually titrated based on tolerability and clinical response, typically reaching 150 mg twice daily (300 mg/day) over 1–2 weeks. For most patients, effective pain control is achieved with total daily doses ranging from 300 to 600 mg. However, some patients may respond well to lower doses, and individualized titration remains essential. Clinical evidence supports the efficacy of pregabalin across a range of neuropathic pain conditions. For example, in postherpetic neuralgia, a dosage of 600 mg/day has demonstrated superior analgesic efficacy compared to lower doses [16], although therapeutic benefit is generally observed within the range of 300–600 mg/day [51]. Similarly, daily doses of 300 mg, 450 mg, and 600 mg are effective in the treatment of painful DPNP, central neuropathic pain, and fibromyalgia, whereas a daily dose of 150 mg is generally insufficient for significant symptom control [118]. Generally, higher dosages of pregabalin are probably unbearable. If the oral dosage was 300–600 mg/day with insufficient pain relief after 2–4 weeks of administration or if intolerability occurs with 75–600 mg/day dosage, then pregabalin treatment should be discontinued [40]. Pregabalin needs to be individualized for patients with different diseases. For patients with shingles, early analgesia prevents PHN. Therefore, the timing of analgesia in patients with shingles is very important. Early use of pregabalin significantly reduced the herpes zoster pain score during the herpes phase, especially within 7 days of the onset of symptoms [119].

In patients with DPNP, an adequate course of medication is required, and the course often takes several weeks for analgesic drugs to achieve a good analgesic effect. In the early stage of starting pregabalin, titration of the drug dose is required; 4–8 weeks of drug treatment is the basic course of treatment, and sometimes it takes longer‐term medication to relieve and control pain.

For cancer‐related neuropathic pain or chemotherapy‐induced pain, pregabalin has demonstrated efficacy as an adjunct to opioid therapy. In this context, pregabalin not only enhances analgesia but also reduces opioid requirements and mitigates opioid‐related side effects, thereby improving overall quality of life. The most commonly reported adverse events associated with pregabalin are somnolence and dizziness. To minimize these, it is critical to initiate treatment with a low dose and titrate cautiously. If adequate pain relief is achieved with a dosage of 300 mg/day, further dose escalation may not be necessary. In patients with severe or refractory pain, a dosage increase to 600 mg/day may be warranted, provided the patient tolerates the treatment.

In patients treated with combined opioid or morphine, the incidence and severity of adverse reactions may increase with the increasing dosage of pregabalin (AUC), where the AUC of pregabalin with opioids was investigated, and the result showed pregabalin alone or with opioids has a similar effect on sleep and pain severity. On the other hand, pregabalin combined with opioids significantly affects fatigue compared to pregabalin alone; therefore, the dose of pregabalin should be adjusted during the treatment, and patients should be carefully monitored for drowsiness, dizziness, and other uncomfortable symptoms.

### 3.2 Safety of the Clinical Medication

#### 3.2.1 Safety and Adverse Reactions of Pregabalin in pNP

Pregabalin reduces neuropathic pain but was initially considered an antiepileptic drug. With the advancement of time, numerous clinical and animal studies evaluated the safety and side effects of pregabalin use. Several studies found greater characteristics of pregabalin for the treatment of neuropathic pain and related diseases. From a correlation analysis of pregabalin with benign CNS and systemic side effects, it showed limited metabolic and teratogenic adverse effects compared with the other anticonvulsants. Besides the higher efficacy of pregabalin in different neuropathic pain diseases, the safety of pregabalin use brings it as the first recommended therapeutic drug for the management of certain neuropathic pain [40]. Nevertheless, pregabalin is not devoid of side effects. Data from clinical trials and preclinical models indicate that approximately 10% of patients experience common adverse events such as dizziness and somnolence. In most cases, these side effects are mild to moderate in intensity and tend to be self‐limiting. When they occur early during treatment initiation, they often resolve spontaneously within the first few days. If they emerge after dose escalation, they typically subside within 2–4 weeks of continued therapy, reflecting the adaptive capacity of the CNS [120, 121].

While pregabalin is more effective than gabapentin in managing neuropathic pain, direct comparisons of their side‐effect profiles are challenging. Variability in study design, particularly the use of flexible dosing regimens in gabapentin trials versus fixed‐dose protocols in pregabalin studies, complicates the interpretation of tolerability outcomes. Despite these methodological differences, most studies report overlapping adverse effects between the two agents, including dizziness, fatigue, and somnolence. However, gabapentin appears to be more frequently associated with gastrointestinal side effects such as nausea and diarrhea [86, 122].

#### 3.2.2 Dosage for a Specific Population

Pregabalin is eliminated primarily as an unchanged drug with less than 2% metabolites by renal excretion. The dosage needs to be adjusted in patients with impaired renal function given dose‐dependent adverse reactions. Dose modification is recommended once creatinine clearance (CLcr) falls below 60 mL/min. For patients with a CLcr of 30–60 mL/min, the total daily dose should generally be reduced by about half relative to individuals with normal renal function (CLcr > 60 mL/min). Additional dose reductions of roughly 50% are advised for every further 50% decline in CLcr (Table 3). Even that pregabalin is efficiently removed during hemodialysis, patients receiving chronic hemodialysis typically require supplemental post‐dialysis dosing to maintain therapeutic plasma concentrations [124]. Furthermore, pregabalin exhibits minimal plasma protein binding, although this characteristic has not been extensively investigated. Therefore, it is unlikely that patients with hepatic impairment require dosing modifications. Since pregabalin can be excreted through human milk, the potential risk outweighing the benefits should be considered. Breastfeeding with pregabalin intake should be cautious, depending on the individual, especially while nursing newborns or infants (LYRICA (pregabalin) oral solution (fda.gov)). In a multiple‐dose, open‐labeled, pharmacokinetic study, 10 healthy lactating women orally received pregabalin with four dosages of pregabalin, each at 150 mg at 12‐h intervals. Geometric mean pregabalin Cmaxss and AUCτ values in breast milk were approximately 53% and 76%, respectively, of plasma values. The estimated average daily infant dosage of pregabalin from breast milk was 0.31 mg/kg/day, which was approximately 7% (23% coefficient of variation) of the body weight normalized maternal dose. Lactation did not meaningfully alter pregabalin disposition in the mothers, and the amount transferred to infants via breast milk was considered low. The medication was also well tolerated by all participating women [125]. Few studies have evaluated the safety of pregabalin use during pregnancy. Across four available studies, no definitive link was identified between prenatal pregabalin exposure and adverse maternal or fetal outcomes. However, these findings remain inconclusive, as the analyses were insufficiently powered to detect potential risks with confidence [126].

**TABLE 3:** Median ratio (children vs. adults) for pharmacokinetic properties of pregabalin exposure based on simulations [123].

		Adults (≥ 17 years)		Children (4–16 years)		
		Median (μg/mL) (*n* = 1000)		All (*n* = 1000)	< 30 kg^a^ (*n* = 391)	≥ 30 kg^b^ (*n* = 609)
Parameter	Frequency	150 mg/day	600 mg/day	Ratio to adults		
*C*_av,ss_ ^c^	BID	1.34	5.37	0.91	0.89	0.92
	TID	1.34	5.35	0.90	0.88	0.91
*C*_max,ss_	BID	2.43	9.73	1.01	1.05	0.99
	TID	1.97	7.86	0.99	1.02	0.97
*C*_min,ss_ ^d^	BID	0.60	2.39	0.75	0.68	0.80
	TID	0.81	3.23	0.78	0.72	0.81

Abbreviations: *C*_av,ss_, average steady‐state concentration; *C*_max,ss_, maximum steady‐state concentration; *C*_min,ss_, minimum steady‐state concentration.

^a^ 3.5 mg/kg/day (max 150 mg/day) or 14 mg/kg/day (max 600 mg/day) for the equivalent adult doses of 150 and 600 mg/day, respectively.

^b^ 2.5 mg/kg/day (max 150 mg/day) or 10 mg/kg/day (max 600 mg/day) for the equivalent adult doses of 150 and 600 mg/day, respectively.

^c^ Ratios to adults are the same as areas under the concentration‐time curve.

^d^ *C*_min,ss_ represents the trough value at 12 h for b.i.d. dosing and 8 h for t.i.d. dosing.

Above all, pregabalin use in pregnancy, lactating women, and nursing newborns or infants was therefore best restricted to circumstances in which the risk–benefit ratio was favorable, after shared decision‐making.

#### 3.2.3 Tapering Off or Reduction of the Dosage

Although tapering‐related symptoms have been reported in isolated cases, current evidence remains insufficient to fully elucidate their underlying mechanisms. These symptoms are generally mild to moderate and typically resolve within approximately 1 week after dose reduction or discontinuation. A 62‐year‐old female who took 150 mg/day of pregabalin reported having anxiety and depression after stopping the medication. Other cases reported different symptoms with pregabalin discontinuation such as tremors, diarrhea, diaphoresis, paranoia, mutism, agitation, hallucination, or suicidal ideas, and increased risk of confusion. Generally, pregabalin discontinuation is safe when the dosage is tapered progressively over 1 week with careful monitoring. Finally, several studies and cases reported the possibility of developing pregabalin addiction and dependence [84, 127, 128, 129]. In addition, Sweet et al. discuss the concerns associated with tapering pregabalin in patients who have a history of substance misuse. The study indicates that the potential for abuse may be significant in this particular group, emphasizing the importance of a cautious approach [130]. A case highlighted by Papazisis et al. [131] of a 19‐year‐old patient exhibiting substance‐seeking behavior towards pregabalin underscores the importance of monitoring and assessing abuse potential, especially during tapering phases. This is particularly critical in individuals with a history of such behavior [131]. The study by Osman and Casey raises concerns about the addictive potential of pregabalin tapering, particularly in cases where individuals experience enhanced effects such as increased sexual desire and excitement. Such factors can contribute to the abuse of pregabalin during the tapering process [132]. In conclusion, while pregabalin serves as an effective therapeutic agent, its tapering process should be handled with utmost care to mitigate the risk of abuse, especially in individuals with a history of substance abuse or dependence.

### 3.3 Prospects of Clinical Application of Pregabalin in the Future

As a first‐line treatment for various neuropathic pain syndromes, pregabalin has firmly established its role in clinical practice. Its efficacy extends beyond trigeminal neuralgia, encompassing both central and peripheral neuropathic conditions. A growing compendium of clinical trials, exploring an array of neuropathic disorders, continues to substantiate the therapeutic benefits of pregabalin. It has been consistently shown to diminish pain intensity across a spectrum of neuropathic pain states and is linked with rapid improvements in sleep quality, often within the first couple of days of treatment. Functional enhancements have also been documented in patients grappling with neuropathic or postoperative pain, aligning the empirical evidence with real‐world clinical outcomes. Nonetheless, the clinical administration of pregabalin necessitates cautious titration, with dosage incrementally adjusted at treatment onset and equally gradual tapering to mitigate side effects. Adverse effects such as dizziness and somnolence warrant vigilant monitoring. Looking to the future, the therapeutic landscape will likely evolve with the introduction and increasing use of mirogabalin, a newer agent that shares a similar mechanism of action with pregabalin but may offer distinct pharmacokinetic advantages [133]. Pregabalin is now one of the first‐line recommended drugs for neuropathic pain, except for trigeminal neuralgia, including central and peripheral neuropathic pain. Many more trials have been completed with some examining different conditions. Pregabalin exerts beneficial effects on some symptoms of neuropathic pain. It can reduce pain intensity in multiple conditions. Apart from controlling pain, pregabalin appears to improve sleep quality in patients with pain disorders within 1–2 days of treatment. It was also associated with functional improvement in patients with neuropathic or postsurgical pain. The evidence in the literature was consistent with the clinical experience (Table 4).

**TABLE 4:** The approved clinical indications of pregabalin in different regions (Pfizer Inc., 2020) and indications for clinical application and dosages.

Conditions	Regions where approved	Initial dosage	Maintenance dosage
Neuropathic pain (including central and peripheral)	Europe, Japan	150 mg/d	300–600 mg/d
Postherpetic neuralgia (PHN)	USA, China	150 mg/d 75–150 mg/d	300–600 mg/d
Fibromyalgia	USA, China	150 mg/d 75–150 mg/d	300–600 mg/d
Diabetic peripheral neuropathy (DPNf)	USA	150 mg/d	300 mg/d
Neuropathic pain associated with spinal cord injury	USA	150 mg/d	300–600 mg/d
Cancer pain and peripheral neuropathy induced by chemotherapy		75–150 mg/d	300 mg/d
Trigeminal neuralgia		75–150 mg/d	300–600 mg/d
Postoperative or post‐traumatic neuralgia		150 mg/d	300–600 mg/d
Traumatic neuroma		150 mg/d	300–600 mg/d

However, it is necessary to pay attention to gradually increasing the dosage of pregabalin at the beginning of taking it, slowly reducing dosage within 1 week instead of sudden tapering of the drug, and observing the patient's dizziness, drowsiness, and other adverse reactions. In the future, clinicians or researchers will perform more high‐quality clinical studies to substantiate its efficacy and safety.

## 4 Statement

We identified six consensus statements regarding the use of pregabalin in peripheral neuropathic pain from a pool of 133 studies. Based on expert ratings, the statements regarding the efficacy and clinical benefits of pregabalin in various neuropathic pain conditions receive varying levels of endorsement (Table 5):

**TABLE 5:** Consensus statement on the use of pregabalin for peripheral neuropathic pain.

	Consensus statements	Level of consensus among experts
Clinical applications statement		
Statement 1	Pregabalin can effectively reduce pain symptoms and improve sleep patterns in patients with PHN, with a good safety level.	100%
Statement 2	There are significant clinical benefits associated with pregabalin in patients with DPN, including stable pain relief, better pain scores and tolerability levels, and anxiety and sleep disturbances.	100%
Statement 3	Patients with advanced cancers have moderate to severe pain lasting months or years and reducing pain and neuropathic symptoms is the primary goal in patients with NCP. As a new generation anticonvulsant, pregabalin is effective in the treatment of neuropathic pain by reducing calcium‐dependent neurotransmitters located on the nerve membrane, is more bioavailable than the other neuromodulators, has no drug interactions, and can be safely used in combination with chemotherapy drugs.	100%
Statement 4	Pregabalin can be used for postoperative neuralgia or post‐traumatic neuralgia. Traumatic neuroma requires reevaluation based on the patient's condition and the physician's experience.	100%
Statement 5	Pregabalin is used to treat patients with TN with few side effects, fewer drug interactions, and significant pain relief at low dosages (150 mg/day) compared to similar drugs, especially in elderly patients.	86%
Statement 6	In the treatment of nerve compression syndromes, such as CTS, pregabalin can be considered as part of the treatment regimen. In addition to pregabalin, it's advisable to prioritize the use of topical pain relief patches, like lidocaine patches, as part of a multimodal pain management approach.	86%

Abbreviations: CTS, carpal tunnel syndrome; DPN, diabetic peripheral neuropathy; NCP, neuropathic pain; PHN, postherpetic neuralgia; TN, trigeminal neuralgia.

Statement 1, rated at 100%, asserts that pregabalin effectively alleviates pain and improves sleep in patients with post‐herpetic neuralgia (PHN), with a high level of safety.

Statement 2, also rated at 100%, highlights significant clinical benefits of pregabalin in diabetic peripheral neuropathy (DPN), including stable pain relief, improved pain scores, and better tolerability, addressing anxiety and sleep disturbances.

Statement 3, also rated at 100%, underscores pregabalin's effectiveness in treating neuropathic pain associated with advanced cancers, emphasizing its mechanism of action in reducing neurotransmitters and its favorable safety profile, particularly when used alongside chemotherapy.

Statement 4, rated at 100%, supports pregabalin's utility in postoperative and post‐traumatic neuralgia, with a caveat on reevaluation for traumatic neuroma based on clinical judgment and experience.

Statement 5, rated at 86%, discusses pregabalin's efficacy in treating trigeminal neuralgia (TN), noting its low dosage requirement and favorable side effect profile, especially beneficial for elderly patients, despite minor concerns over its overall effectiveness.

Statement 6, also rated at 86%, suggests pregabalin as a potential treatment option for nerve compression syndromes like carpal tunnel syndrome, advocating for a multimodal approach that includes topical pain relief patches.

In conclusion, the expert consensus strongly supports pregabalin's efficacy across various neuropathic pain conditions, highlighting its role in pain management with favorable safety profiles in most scenarios. The ratings reflect robust endorsement for its use in PHN, DPN, and cancer‐related neuropathic pain, while also acknowledging nuanced considerations in conditions like TN and nerve compression syndromes. These findings underscore pregabalin's versatility and reliability in clinical practice for managing neuropathic pain.

## Author Contributions

Qingwei Zhao, Yihe Hu, designed the study. Jian Wang, Wan Shuanglin, Du Dongping, MA Ke, Zhiying Feng, Suming Tian, and Weidong Qiu; Kaiming Liu, Li Lin, Xianfeng Zhang, Gong Liyan, Haiqi Lu, and Yan Liu analyzed the results. Hui Lu, Yuefeng Rao, Xu Jin, Zhongwei zhao, Zhenhua Zeng, Benyan Luo, Changqing Wang, and Hui Zhao drafted the manuscript. The authors have read and approved the final manuscript.

## Funding

The authors have nothing to report.

## Disclosure

Contribution to the Field Statement: The study analyzed the clinical use of pregabalin in different diseases that are associated with peripheral neuropathic pain.

## Ethics Statement

The authors have nothing to report.

## Conflicts of Interest

The authors declare no conflicts of interest.

## Data Availability

The authors have nothing to report.
